# Immunoregulatory properties of rapamycin-conditioned monocyte-derived dendritic cells and their role in transplantation

**DOI:** 10.1186/2047-1440-1-16

**Published:** 2012-09-28

**Authors:** Camila Macedo, Hēth Turquist, Diana Metes, Angus W Thomson

**Affiliations:** 1 Thomas E. Starzl Transplantation Institute, Department of Surgery, University of Pittsburgh School of Medicine, 200 Lothrop Street, Pittsburgh, PA 15261, USA; 2 Department of Immunology, University of Pittsburgh School of Medicine, 200 Lothrop Street, Pittsburgh, PA 15261, USA

**Keywords:** Dendritic cells, Antigen presentation, Rapamycin, T cells, Regulatory T cells, Tolerance, Transplantation

## Abstract

In efforts to minimize the chronic administration of immunosuppression (IS) drugs in transplantation and autoimmune disease, various cell-based tolerogenic therapies, including the use of regulatory or tolerogenic dendritic cells (tolDC) have been developed. These DC-based therapies aim to harness the inherent immunoregulatory potential of these professional antigen-presenting cells. In this short review, we describe both the demonstrated tolerogenic properties, and current limitations of rapamycin-conditioned DC (RAPA-DC). RAPA-DC are generated through inhibition of the integrative kinase mammalian target of rapamycin (mTOR) by the immunosuppressive macrolide rapamycin during propagation of monocyte-derived DC. Consistent with the characteristics of tolDC, murine RAPA-DC display resistance to phenotypic maturation induced by pro-inflammatory stimuli; exhibit the ability to migrate to secondary lymphoid tissue (important for ‘cross-presentation’ of antigen to T cells), and enrich for naturally-occurring CD4^+^ regulatory T cells. In rodent models, delivery of recipient-derived RAPA-DC pulsed with donor antigen prior to organ transplantation can prolong allogeneic heart-graft survival indefinitely, especially when combined with a short course of IS. These encouraging data support ongoing efforts to develop RAPA-DC for clinical testing. When compared to murine RAPA-DC however, human RAPA-DC have proven only partially resistant to maturation triggered by pro-inflammatory cytokines, and display heterogeneity in their impact on effector T-cell expansion and function. In total, the evidence suggests the need for more in-depth studies to better understand the mechanisms by which mTOR controls human DC function. These studies may facilitate the development of RAPA-DC therapy alone or together with agents that preserve/enhance their tolerogenic properties as clinical immunoregulatory vectors.

## Introduction

Given their capacity to safely prevent and/or reverse acute allograft rejection, immunosuppressive agents have proven crucial to the successful clinical development of organ transplantation. However, there are major limitations associated with drug-based immunosuppression (IS), including lack of antigen (Ag) specificity, failure to support tolerance induction, deficiencies in the prevention of late graft failure (chronic rejection), and significant morbidity. Evaluation of novel, tolerance-promoting protocols, including cell-based therapies, such as the use of tolerogenic dendritic cells (tolDC), is a dynamic area of investigation and may provide a means to minimize or even replace use of IS drugs [1]. In addition to reducing the toxic burden of chronic IS, it is hoped that these innovative approaches will prevent/reduce chronic rejection, given the strong immunological involvement in its etiology [2,3].

DC play critical roles in Ag presentation to naïve and memory T cells and can either promote T-cell immunity or support the induction of tolerance [4-6]. Experimental protocols are currently being developed with the goal of harnessing the inherent tolerogenicity of DC to act as ‘negative cellular vaccines’, which can inhibit immune responses in an alloAg-specific manner and promote tolerance to transplanted cells and organs. TolDC are characteristically immature, express low surface MHC molecules, a low ratio of co-stimulatory to co-inhibitory signals, and an impaired ability to secrete T-helper-1 (Th1) cell-driving or inflammatory cytokines [2].

In particular, administration of tolDC presenting allo-Ag promotes transplant survival through the induction of T-cell hyporesponsiveness to allo-Ag, deletion of alloreactive T cells, or increased Treg incidence or functions [7-10]. This knowledge has driven efforts to identify mechanisms that underline the tolerogenic properties of DC to promote transplant tolerance.

Various anti-inflammatory and immunosuppressive agents have been used to generate tolDC *in vitro*, including interleukin (IL)-10 and transforming growth factor-β (TGF-β), cytotoxic T lymphocyte Ag-4 Ig (CTLA4-Ig), prostaglandin E2 (PGE2), dexamethasone, and vitamin D3 (vitD3), among others [11-13]. Of special interest to our lab has been defining the molecular and functional impact of the immunosuppressive pro-drug rapamycin (RAPA) on DC.

### Phenotypic and functional characteristics of RAPA-DC

RAPA is a macrocyclic triene antibiotic with immunosuppressant properties, that was discovered in 1975 as a product of the bacterium *Streptomyces hygroscopicus* in a soil sample from Rapa Nui (Easter Island) [14]. This immunosuppressant inhibits the mammalian target of rapamycin (mTOR), a highly conserved serine/threonine kinase that controls cellular responses to environmental cues [15-17]. In mouse models, RAPA has a profound impact on DC *in vitro*, impairing their maturation following exposure to Toll-like receptor (TLR) ligands and suppressing their T-cell allostimulatory function [11,18-22] (Table 1). RAPA has been reported to have unique tolerance-promoting and Treg facilitating/sparing properties in small animal models of organ transplantation [23,24]. We [10,25] and others [7,21,26,27] have shown that when donor-derived, RAPA-conditioned DC (RAPA-DC) or recipient-derived RAPA-DC pulsed with donor allo-Ag are administered to recipients prior to transplantation, donor graft survival is prolonged indefinitely, especially when combined with a short course of low dose IS, such as RAPA, cyclosporine, or FK506 (Table 2). Taner *et al.* have shown, in the mouse model, prolongation of heart allograft survival when recipient-derived RAPA-DC pulsed with allo-Ag were given i.v. prior transplantation. Such results were improved with short-term administration of subtherapeutic dose FK506, which alone did not prolong graft survival or repeated infusion of RAPA-DC pulsed with allo-Ag (x3; days −10, -3, and 0) [10]. Turnquist *et al.* have also shown long-term heart allograft survival, after a single i.v. dose of recipient-derived RAPA-DC pulsed with alloAg (day −7) followed by a short-term course of low-dose RAPA [25].

**Table 1:** **Mouse *vs. *human immature RAPA-DC**

	**Mouse**	**Human**
**Phenotype**[19,23,26,27]		
MHC Class II	↓	-
CD86/ CD40	↓↓	↓
B7-H1	↓	↓
**Migration**[10,23,24]		
CCR7	-	↑
**Treg**[21-23]		
enrichment/induction/expansion	↑	↑
**T cell apoptosis**[10]	**↑**	**ND**
**Function**[15,19,20,23,27-29]		
Endocytosis	↓↓	ND
MLR	↓↓	↓
IL-12p70	↓	-
IL-10	↓	-

ND, not done; -, not changed.

**Table 2:** Prolongation of allograft survival by RAPA-DC

**Species**	**Graft**	**Source of DC**	**IS agent *in vivo***	**Reference**
Mouse	Heart	Bone marrow (BM) recipient-derived	None	10
			FK506	10
			RAPA	23
Rat	Limb	BM recipient-derived	ALS, CSA	18
Rat	Vascularized skin	BM recipient-derived	ALS, CSA	7
Mouse	Hematopoietic cells	Autologous BM-derived (unpulsed)	None	24
Mouse	Pancreatic islets	BM donor-derived	None	25

ALS, anti-lymphocyte serum; CSA, cyclosporine A; IS, immunosuppression.

When rodent and human DC are generated in clinically-relevant concentrations of RAPA, they are phenotypically immature, with low levels of cell surface T-cell co-stimulatory molecules (CD86, CD40); however, only murine RAPA-DC maintain their immature phenotype when exposed to inflammatory stimuli, such as bacterial lipopolysaccharide (LPS) [11,25]. Also, mouse and human RAPA-DC exhibit a paradoxical decrease in cell surface expression of B7-H1 (also known as programmed death ligand-1; PD-L1), a PD-1 ligand, which contributes to the negative regulation of T lymphocyte activation and promotes peripheral tolerance [13,28] (Table 1).

Murine RAPA-DC induce hyporesponsiveness and/or apoptosis of alloreactive T cells [10,11,18,22,25]. Likewise, human RAPA-DC are poorly stimulatory and induce T-cell hyporesponsiveness [11]. Furthermore, murine RAPA-DC retain the capacity to stimulate mouse naturally-occurring Foxp3^+^ Treg, resulting in an overall enrichment of this population relative to T effector cells [25]. A similar capacity for promotion of Foxp3^+^ cells in T-cell cultures has been reported for human RAPA-DC [12] (Table 1).

Another important feature of murine RAPA-DC is their unaltered chemokine receptor (CCR7) expression and capacity for migration to CCL19/CCL21, and thus to secondary lymphoid tissues [10,25,26]. Human RAPA-DC have been reported to upregulate CCR7 expression and to display significantly augmented migration to CCL21 compared to control DC or other *ex vivo*-generated human tolDC, such as those conditioned with IL-10, dexamethasone, TGF-β, or vitamin D3 [13,30]. The ability of RAPA-DC to retain CCR7 expression/regulation and to migrate *in vivo* to secondary lymphoid tissue, while maintaining low expression of CD86 and diminished T-cell allostimulatory capacity, has important implications for their function as cellular therapy (that is, ‘negative’ vaccines) for prevention of transplant rejection [31] (Table 1).

RAPA-DC are also characterized by their unique cytokine production profile upon LPS or pro-inflammatory cocktail (IL-1β, tumor necrosis factor (TNF)-α, IL-6, IFN-γ) stimulation. While IL-10 production is consistently reduced in RAPA-DC [12,28], their production of IL-12p70 may be affected differently. DC exposed to RAPA *in vivo* exhibit decreased IL-12p70 production in response to IL-4 stimulation; likewise, when DC are generated in culture with long exposure to RAPA, followed by stimulation with agonistic anti-CD40 mAb, these RAPA-DC display reduced IL-12p40 [18,25]. However, we have described increased IL-12p70 production by human monocyte-derived RAPA-DC after stimulation with LPS [11,29] or pro-inflammatory cytokines (Macedo *et al.*, manuscript in preparation). Increased production of IL-12p70 by RAPA-DC upon maturation (LPS stimulation) has been associated with augmented Th1/Th2-polarization of alloreactive CD4^+^ T cells [32] and with Th1 responses upon pro-inflammatory cytokine stimulation, towards IFN-γ production (Macedo *et al.*, manuscript in preparation). Further definition of the precise mechanisms by which mTOR controls and coordinates cytokine production and expression of B7-H1 by DC upon exposure to pro-inflammatory stimuli will be important to fundamental understanding of DC immunobiology and aid efforts to harness these promising immunoregulatory vectors in transplant medicine and autoimmune disease.

### RAPA-DC from the bench to the clinic

The use of immunogenic or tolDC-based cell therapy in the clinic has been reported by groups working in different medical fields (cancer, HIV infection, and autoimmune diseases) with positive outcomes in terms of its feasibility and safety [33-36]. One aspect of tolDC-based cell therapy in organ transplantation involves the use of donor-derived tolDC in an effort to improve graft survival; however, such protocols can only be applied in a live-donor setting since the *in-vitro* generation of tolDC takes 5 to 7 days, precluding use of tolDC generated from deceased donors. The generation of recipient-derived DC loaded with donor allo-Ag (donor cell lysate, apoptotic cells, or exosomes) is more advantageous, since the generation of autologous RAPA-DC can be performed at any time before transplantation and host peripheral mononuclear cells (PBMC) can be cryopreserved until time of tolDC generation/infusion. In addition, Ag presentation via the indirect pathway is thought to play an important role in the development of chronic rejection, making recipient-derived DC, if successful in regulating indirectly-alloreactive T cells, a potentially ground-breaking tolerogenic cell therapy in transplantation [37]. Immature DC such as RAPA-DC can also regulate the expansion and differentiation of Treg *in vitro* and *in vivo*, resulting in a ‘feedback’ regulatory loop [38,39]. On the current evidence, we cannot say whether pre- or post-transplant administration of tolDC, or whether autologous or donor-derived tolDCs, will prove to be a superior treatment; however, it is our personal opinion that alloantigen-pulsed recipient-derived DCs represent a pragmatic approach and offer certain theoretical advantages because of their indirect presentation of alloantigen. It is an exciting prospect that The ONE Study consortium will directly compare different approaches to tolerogenic APC therapy in a coherent clinical trial.

A means to obtain large numbers of monocytes is through their enrichment from peripheral blood leukapheresis products. The Elutra^TM^ cell separation system enriches monocytes untouched by antibodies or microbeads within a closed system on the basis of size and density [35,36]. Although not yet approved for clinical use in many countries, as an alternative, the CliniMACS® cell separation system isolates monocytes by positive selection using CD14 microbeads within a closed system with a good purity [40,41]. Monocytes generated using these techniques can be cultured in medium containing cGMP-grade GM-CSF and IL-4, or using an Aastrom Replicell system [12,35]. After 5 to 7 days of incubation, DC can be loaded with allo-Ag, then evaluated for sterility, viability, recovery, and phenotype; and either aliquoted for cryopreservation or infused into the patient. We envisaged that the addition of RAPA during human DC culture/expansion would promote the tolerogenic features described above for murine RAPA-DC (Table 3). However, exposure of human RAPA-DC to maturation-inducing factors, such as pro-inflammatory cytokines or TLR4 ligands *in vitro* increased their production of IL-12p70, a Th1-inducing cytokine that could augment pathogen-specific CD8^+^ T cell responses and/ or promote alloimmunity [11,42] and (Macedo *et al.*, manuscript in preparation). As such, methods to limit IL-12p70 production should be examined as part of any protocol for RAPA-DC generation [11,43-45]. Recently, we have shown that increased IL-12p70 production, by both mouse and human RAPA-DC, following TLR4 ligation results from lost regulation of glycogen synthase kinase 3 (GSK-3) [11]. As treatment of RAPA-DC with GSK-3 inhibitors, such as lithium chloride, ablated IL-12p70 production, RAPA-DC treatment with GSK-3 inhibitors may be useful in limiting any potential danger of increase Th1 immunity following RAPA-DC administration [11].

**Table 3:** Generation of immature RAPA-DC

	**Research**	**Clinical**
Monocyte isolation	CD14 microbeads (AutoMACS® or CliniMACS®)	Elutriation or CD14 microbeads (CliniMACS®)
Culture conditions	10% fetal calf serum	10% human serum
(7 days)	AIM-V media	AIM-V media (c-GMP grade)
	1000 U/mL rhIL-4	1000 U/mL rhIL-4 (c-GMP grade)
	1000 U/mL GM-CSF	1000 U/mL GM-CSF (c-GMP grade)
	10 ng/mL Rapamycin	10 ng/mL Rapamycin (c-GMP grade)
	Cell culture plates	Cell culture plates or Aastrom Replicell system

Another way to maintain RAPA-DC tolerogenicity following infusion of donor-derived or allo-Ag-pulsed recipient-derived tolDC is the concomitant use of costimulation-blocking agents, such as abatacept (CTLA4-Ig) or belatacept (Lea 29Y), a first and second-generation CTLA4-Ag, respectively, that block the B7-CD28 costimulatory pathway [46,47]. Lu *et al.*[48] showed an increase in experimental organ graft survival when anti-CD40L mAb was administered in conjunction with donor-derived myeloid DC to block the CD40/CD40L pathway, which plays an important role in allogeneic DC-T cell interactions *in vivo*. Later, Kirk *et al*. [49] and Kenyon *et al*. [50] showed promising results in renal and pancreatic islet transplantation, respectively, following the administration of humanized CD154-specific monoclonal antibody in rhesus monkeys with acute rejection-free and prolongation of graft survival. However, in the following year, Kawai *et al*. [51] documented a high incidence of thromboembolic complications after the use of monoclonal antibody against CD40L in monkeys. Nevertheless, further studies and pre-clinical evaluation of CD40-CD40L pathway blockade in conjunction with tolDC should be explored, including use of anti-CD40 prior to its application in patients.

A further clinically applicable question concerning tolDC therapy is the route of DC administration, since it could promote different outcomes. Giannoukakis *et al.*[36] have reported injection of autologous NF-κB-inhibited DC intradermally in the abdominal wall overlying the anatomic location of the pancreas in type-1 diabetic patients. As previously described by our group, in the mouse model, systemic (intravenous) administration of RAPA-DC was successful in significantly prolonging alloAg-specific heart graft survival [10]. Macatangay *et al*. found no difference between subcutaneous and intravenous administration of autologous monocyte-derived DC loaded with HIV-1 peptides delivered to subjects with chronic HIV-1 infection on antiretroviral therapy [52]. Since CCR7 and CD62L expression on RAPA-DC is not affected by mTOR inhibition, this may allow the cells to traffic normally to secondary lymphoid tissues, where their immunoregulatory function is mediated [10,26,30].

### How studies of human RAPA-DC relate to others’ work

In recent years, tolDC protocols have offered a potential therapeutic tool in solid organ transplantation [1,2,53]. In order to compare different tolDC protocols, specific characteristics of the tolDC need to be analyzed. These include the phenotype, migration capability, cytokine production (in both immature and mature states), the ability to induce allogeneic T-cell proliferation, and the expansion/induction of Treg. For the purpose of generating tolDC, these can be manipulated *in vitro* with different immune modulators such as RAPA, dexamethasone, IL-10, TGF-β, or vitD3 [1,2,11-13].

In humans, the majority of the tolDC generated using the protocols mentioned above exhibit an immature to semi-mature cell surface phenotype, with low to intermediate expression of MHC II, CD86, CD83, and B7-H1. RAPA-DC and TGF-β-DC have a higher migration capability in response to CCL19 and CCL21 *in vitro* when compared to IL-10- and vitD3-DC, with higher expression of CCR7. Interestingly, all tolDC (dexamethasone-, IL-10-, RAPA-, TGF-β-, and VitD3-DC) exhibit diminished production of IL-23 when compared to mature untreated-DC, whereas IL-10-DC and dexamethasone-DC were the only population to show increased production of IL-10 [11-13]. However, the ability of tolDC to suppress T-cell proliferation in humans is variable. IL-10-, TGF-β-, and VitD3-DC can each suppress T-cell proliferation [11-13]. Contradictory effects of RAPA on DC in culture have been reported. We have shown allo-PBMC hyporesponsiviness in MLR when stimulated with RAPA-DC [11]. Naranjo-Gomez *et al*. [12] have also shown RAPA-DC suppression of T-cell proliferation in CFSE-MLR on the other hand, Boks *et al*. [13] did not find RAPA-DC to be suppressive in MLR and Haidinger *et al*. found an allostimulatory effect of RAPA on DC (augmentation of IL-12, CD86, IL-1β, and Ag presentation) [28]. Interestingly, Naranjo-Gomez *et al.* have shown, in humans, as we reported previously in mice, that RAPA-DC are capable of significantly sparing/expanding Treg, which suppress effector T-cell allo-reactivity [11,12].

Other immunosuppressive cell types of myeloid origin, such as macrophages [54], myeloid-derived suppressor cells generated in the presence of PGE2 [55], and mesenchymal stem cells [56], have been introduced recently to the transplant field, with suppressive properties that may be suitable for clinical use. DC treated with different cytokines and/or IS agents, macrophages, and mesenchymal stem cells mentioned above are being studied currently by The ONE Study, a multinational clinical assessment of immunomodulatory cell therapy in renal transplantation [57-59].

## Conclusions

In an effort to reduce the adverse side effects of chronic IS after organ transplantation, tolDC protocols have been used to generate ‘negative cellular vaccines’ with potential therapeutic applicability. We have standardized culture conditions to generate human monocyte-derived RAPA-DC that exhibit tolerogenic characteristics, including a more immature phenotype when compared to control untreated DC. However, human RAPA-DC are not fully resistant to maturation, but can induce Treg, and have potential migratory capacity to secondary lymphoid tissue (spleen and lymph nodes). Although RAPA-DC exhibit unique immunoregulatory properties, the immediate clinical implementation of RAPA-DC is complicated by a dysregulation of pro- *vs.* anti-inflammatory cytokine production, particularly IL-12p70 and IL-10. However, methods to prevent increased IL-12p70 production by RAPA-DC (such as use of lithium chloride [11] or sanglifehrin A [60]) have been identified. Likewise, IL-10 could be delivered with vaccination to offset the reduced capacity of RAPA-DC to make IL-10. Further insights into how mTOR regulates DC cytokine production are critical for development of improved ‘negative’ and ‘positive’ cellular vaccines in general, and to begin to translate these technologies to the bedside.

## Abbreviations

Ag: Antigen; CTLA4-Ig: Cytotoxic T lymphocyte Ag-4 immunoglobulin; DC: Dendritic cells; GM-CSF: Granulocyte macrophage colony stimulating factor; GSK3: Glycogen synthase kinase 3; IL: Interleukin; IS: Immunosuppression; LPS: Lypopolysaccharide; MLR: Mixed leukocyte reaction; mTOR: Mammalian target of rapamycin; PBMC: Peripheral blood mononuclear cells; PGE2: Prostaglandin E2; RAPA: Rapamycin; TGFβ1: Transforming growth factor β1; TLR: Toll-like receptors; TolDC: Tolerogenic dendritic cells; Treg: Regulatory T cells; VEGF: Vascular endothelial growth factor.

## Competing interests

The authors declare that they have no competing interests.

## Authors’ contributions

CM wrote the manuscript. HT, DM, and AWT participated in editing the final manuscript. All authors read and approved the final manuscript.
